# Early Growth Response Protein 1 Promotes Restenosis by Upregulating Intercellular Adhesion Molecule-1 in Vein Graft

**DOI:** 10.1155/2013/432409

**Published:** 2013-12-09

**Authors:** Kui Zhang, Jian Cao, Ran Dong, Jie Du

**Affiliations:** ^1^Cardiac Surgery, Beijing Institute of Heart, Lung and Blood Vessel Diseases, Beijing Anzhen Hospital Affiliated with Capital Medical University, Beijing 100029, China; ^2^Vessel Biology, Beijing Institute of Heart, Lung and Blood Vessel Diseases, Beijing Anzhen Hospital Affiliated with Capital Medical University, Beijing 100029, China

## Abstract

*Objectives*. To verify the relationship between Egr-1 and vein graft restenosis and investigate the related mechanisms. *Methods*. Mouse vein graft models were established in Egr-1 knockout (KO) and wild-type (WT) mice. The vein grafts in the mice were taken for pathological examination and immunohistochemical analysis. The endothelial cells (ECs) were stimulated by using a computer-controlled cyclic stress unit. BrdU staining and PCR were used to detect ECs proliferation activity and Egr-1 and ICAM-1 mRNA expression, respectively. Western-blot analysis was also used to detect expression of Egr-1 and intercellular adhesion molecule-1 (ICAM-1) proteins. *Results*. The lumens of vein grafts in Egr-1 KO mice were wider than in WT mice. ECs proliferation after mechanical stretch stimulation was suppressed by Egr-1 knockout (*P* < 0.05). Both in vein grafts and ECs from WT mice after mechanical stretch stimulation, mRNA expression and protein of Egr-1 and ICAM-1 showed increases (*P* < 0.05). However, ICAM-1 expression was significantly suppressed in ECs from Egr-1 knockout mice (*P* < 0.05). *Conclusions*. Egr-1 may promote ECs proliferation and result in vein graft restenosis by upregulating the expression of ICAM-1. As a key factor of vein graft restenosis, it could be a target for the prevention of restenosis after CABG surgery.

## 1. Introduction

Coronary artery bypass graft (CABG) is one of the most effective therapies for coronary artery diseases (CAD). However, some studies revealed that about 10% of vein grafts occluded within one month after CABG and that only 50% of vein grafts remained unobstructed 10 years after CABG [1]. Thus, how to improve the efficiency of vein grafts over time is a challenge in cardiac surgery. Some of the mechanisms involved in the occlusion of vein grafts have been identified.

Normal vascular endothelial cells (ECs) play a central role in regulating intimal growth [2, 3]. After CABG, vein grafts walls sustain a blood pressure that is much higher than the usual venous pressure, and the walls of the vein graft are often injured by pulsatile stretching [4–6]. These stresses lead to endothelial dysfunction, which is the initial factor inducing the proliferative thickening of the intima [7]. These injuries induce changes in ECs proliferation, cytokines secretion, platelet aggregation, and leukocyte adherence, which are involved in the onset of acute thrombosis [7, 8]. This impairment in the function of ECs then activates vascular smooth muscle cells (VSMCs) migration to the intima, where they transform into a proliferative phenotype. Synthesis and accumulation of an extracellular matrix by these activated SMCs form a matrix for atherosclerosis development [9, 10]. Finally, activated ECs and SMCs produce a number of attracting chemokines, recruiting and retaining monocytes into the endothelium and the extracellular matrix, where they transform into macrophages participating to atherosclerotic plaque development [9, 10]. Together, these three mechanisms are involved in the proliferative thickening of the intima and, ultimately, in CABG failure [11, 12].

Recent studies suggested that three families of adhesion factors are involved in restenosis: (1) the immunoglobulin superfamily (including the intercellular adhesion molecule-1 (ICAM-1) and the vascular cell adhesion molecule-1 (VCAM-1), [13–15]) (2) the integrin family (including the macrophage 1 antigen (MAC-1) and the lymphocyte function associated antigen-1 (LFA-1)) [16, 17], (3) the selectin family (including E-selectin) [18]. Increases in ICAM-1 expression are characteristic of early endothelial dysfunction induced by blood flow mechanical shear stress [19, 20]. ICAM-1 and VCAM-1 participate to lymphocyte attraction and retention, in VSMCs migration and proliferation and in formation of the atherosclerotic plaque [21, 22]. sICAM-1 levels are inversely correlated with endothelial dysfunction [23]. Recently, some studies suggested that sICAM-1 could be used as a biomarker predicting secondary cardiovascular disease in patients with CAD [24, 25].

Early growth response protein 1 (Egr-1) is a member of the immediate early gene family and is also an important transcription factor. Recent studies show that Egr-1 is involved in the regulation of intracellular signaling and multiple genes including growth factor, signal transduction genes, transcription factor, and oncogenes. Other studies also revealed that Egr-1 plays an important role in cell growth, development, differentiation and wound healing [26–28]. Our preliminary study showed that Egr-1 is closely related with restenosis, and the degree of vein graft stenosis in Egr-1 knockout (KO) mice was significantly lower than that in wild-type (WT) mice, indicating that Egr-1 could promote vein graft stenosis. However, the mechanisms involved in this effect of Egr-1 on vein graft stenosis were unclear. To our knowledge, no such information has been reported. Therefore, this study further verified the effect of Egr-1 on vein graft restenosis and investigated the related mechanisms. We hoped that this study would provide valuable information for prevention and treatment of vein graft restenosis.

## 2. Materials and Methods

### 2.1. Animals

We used male Egr-1 KO mice as described [29] and WT C57BL/6J mice (Vital River Company, Beijing, China), aged between 8 and 12 weeks. All animals were managed according to the guidelines of Beijing An Zhen Hospital, Capital Medical University. Experimental protocols were approved by Cardiac Surgery of Beijing An Zhen Hospital, Capital Medical University. Before experiments, mice were kept for one week at 24°C, on a 12-hour light/dark cycle and received a normal diet. In the experimental group, Egr-1 KO mice were used as donors, and littermate Egr-1 KO mice were used as recipients. In the control group, WT C57BL/6J mice were used as donors, and littermate WT C57BL/6J mice were used as recipients. Egr-1 KO mice originated from a C57BL/6J × 129 background and were back-crossed with the C57BL/6J strain for at least 10 generations. Animal experiments were approved by the Beijing Institute of Heart, Lung and Blood Vessel Diseases.

### 2.2. Mouse Vein Graft Model

We used a vein graft model as previously described by Zou et al. [30]. In brief, mice were anesthetized using an intraperitoneal injection of 1% pentobarbital sodium. The right common carotid artery was dissected from surrounding tissue as far (distally and proximally) as possible. We ligatured the right common carotid artery at midpoint with sutures and divided it. Distal and proximal segments were combined inside a cuff, and we controlled the arterial inflow and outflow using clamps placed at the cuffs' handles. Sutures were removed; distal and proximal arteries were everted over the cuffs' tubular body and fixed using sutures. Consequently, a sleeved vein segment (inferior vena cava from donor mice) was grafted over the cuffs and secured into position with sutures. Finally, incision was closed using 3-0 Dacron (Hangzhou AiPu Medical Products Co., Zhejiang, China).

We tested the patency of vein graft after the operation. If the grafted vessel pulsed vigorously and there was no bleeding, we considered that the surgery was a success. If there was no pulsation within a few minutes of blood flow restoration or if clot formation was suspected, the surgery was considered a surgical failure [30]. We harvested the vein grafts at 3 h, 1 day, 1 week, 2 weeks, and 4 weeks after surgery.

Mice in the sham surgery group (sham) were anesthetized and their neck was incised, but no surgery was performed on the carotid arteries.

### 2.3. Mechanical Stretch Stimulation

ECs isolated from the inferior vena cava endothelial cells (IVCECs) and vena cava of Egr-1 KO and WT mice were kept in Dulbecco's modified Eagle medium (DMEM) with 10% fetal bovine serum (FBS) at 37°C with 95% air and 5% CO_2_. We then plated ECs on silicone elastomer-bottomed and collagen-coated plates (Flexcell, McKeesport, PA, USA). A computer-controlled cyclic stress unit (Flexcell, McKeesport, PA, USA) was used to subject ECs to mechanical stretch (cyclic deformation was 60 cycles/min, elongation was 15%) [31, 32].

### 2.4. Measure of ECs Proliferation

We performed BrdU immunostaining according to the manufacturer's protocol (Roche Molecular Systems, Pleasanton, CA, USA). In brief, ECs were cultured for 12 hours in medium containing 0.4% FBS to synchronize them, then cultured in DMEM with 10% FBS, and stimulated with mechanical stretch for 24 hours. In the last 4 hours of cell culture at 37°C, BrdU was added. After being washed three times with PBS, cells were fixed using 4% paraformaldehyde for 10 min, washed three times with PBS, exposed to 0.3% Triton X-100 for 5 min, and washed three times with PBS again. Cells were treated with 2 M HCl at 37°C for 30 min, then with 0.1 M sodium tetraborate for 10 min, and washed three times with PBS. Cells were incubated in 10% fetal calf serum for 30 min before being incubated overnight at 4°C with antimouse BrdU monoclonal antibody. Finally, the cells were treated with fluorescence-labeled goat antimouse IgG at room temperature for 1 h. PBS was used as a negative control. DAPI was used for staining nuclei specifically. The mean percentage of cells positive for BrdU was determined in 4 or 5 different fields using a Leica DMI4000B fluorescence microscope (Leica, Wetzlar, Germany) under 100× magnification. Count was repeated 4 times.

### 2.5. Real-Time RT-PCR

Total RNA was extracted from ECs stimulated with mechanical stretch, using Trizol reagent (Sigma, Saint Louis, MO, USA). RNA purity was determined using absorbance at 260 and 280 nm (A260/280). After assessing RNA concentration, total RNA was reverse-transcribed into complementary DNA (cDNAs) using a first strand cDNA synthesis kit (Fermentas Life Sciences). For mouse Egr-1 amplification, primers were forward, 5′-CAG CAG CCT TCG CTA ACC-3′, and reverse, 5′-CCA CTG GGC AAG CGT AA-3′. For mouse ICAM-1 amplification, primers were forward, 5′-AGG TGT GAT ATC CGG TAG AT-3′ and reverse, 5′-CCT TCT AAG TGG TTG GAA CA-3′. were forward, 5′-TGA CTT CAA CAG CGA CAC CCA-3′, Primers for mouse GAPDH and reverse, 5′-CAC CCT GTT GCT GTA GCC AAA-3′. All reactions involved initial denaturation at 95°C for 15 s, followed by 45 cycles of 95°C for 5 s and 60°C for 30 s. Specific mRNA quantification was performed by real-time PCR using SYBR Premix Ex Taq II (TaKaRa Bio, Dalian, China) in a TP800 real-time PCR System (TaKaRa Bio, Dalian, China), according to the guidelines provided by the manufacturer. Results were analyzed using the comparative threshold cycle, as previously described [33].

### 2.6. Western-Blot Analysis

ECs from the vena cava of Egr-1 KO and WT mice were stimulated with mechanical stretch, then lysed in RIPA buffer (50 mmol/L Tris, 150 mmol/L NaCl, 5 mmol/L EDTA, 1% NP-40, 1% sodium deoxycholate, 0.1% SDS, 1 mmol/L Phenylmethanesulfonyl fluoride (PMSF), 10 ug/mL aprotinin, 1 mmol/L NaVO_4_), and centrifuged 15 min at 4°C (12000 rpm). After protein concentration was measured using the BCA protein assay kit (HyClone-Pierce, Utah, CA, USA), equal amounts of protein (40 *μ*g) were separated on 8% SDS-polyacrylamide gels and transferred onto PVDF membranes (Millipore, Massachusetts, CA, USA). The nonspecific sites on each blot were blocked with 5% nonfat milk powder diluted in TBS with 0.05% Tween 20 (TBST). Membranes were incubated overnight with primary antibodies against IL-1, IL-4, TGF-*β*, and TNF-*α* (Abcam, Cambridge, UK) at 4°C. After washing by TBST, proteins were revealed with HRP-labeled IgG (Pierce antibodies, Thermo Fisher Scientific, Waltham, MA, USA) at room temperature for 1 hour. Membranes were developed using the ECL system (Amersham) and analyzed using the Biological electrophoresis image analysis system (Furi Science & Technology Company, Shanghai, China).

### 2.7. Immunohistochemistry

Immunohistochemistry was performed on 4 *μ*m thick formalin-fixed, paraffin-embedded tissue sections. Deparaffinized sections were treated with 3% hydrogen peroxide for 30 min at 30°C and boiled in 1% citric acid solution for 20 min after washing in PBS. After blocking with 10% sheep serum (Vector Laboratories, Burlingame, CA, USA) for 30 min, sections were incubated with the primary antibody against Egr-1 (Epitomic, San Francisco, CA, USA) and ICAM-1 (Abcam, Cambridge, UK) overnight. After washing with PBS, sections were incubated with the secondary antibody (goat antirabbit, Vector Laboratories, Burlingame, CA, USA) for 40 min at 30°C, and finally with avidin-biotin complex (Vector Laboratories, Burlingame, CA, USA) for 10 min. Immunogenicity was visualized using 3,3′-diaminobenzidine (Vector Laboratory, Burlingame, CA, USA) for 2 min. Sections were counterstained with hematoxylin, dehydrated in alcohol, and cleared with Histo-clear. For negative control, the same protocol was used with antigen dilution reagent instead of the primary antibodies. Stained slides were viewed with light microscope, images were captured, and the percentages of positive cells in vein grafts were analyzed using the NIS Elements BR software (Nikon Instruments Inc., Japan).

### 2.8. Statistical Analysis

Results are presented as mean ± SEM. Comparisons between groups were analyzed by one-way ANOVA followed by the Bonferroni/Dunn post hoc analysis. All statistical analyses were performed using SPSS 17.0 (SPSS Inc., Chicago, IL, USA). A *P* value <0.05 was considered statistically significant.

## 3. Results

### 3.1. Egr-1 KO Decreases Stenosis in Vein Grafts

**Figure 1:**
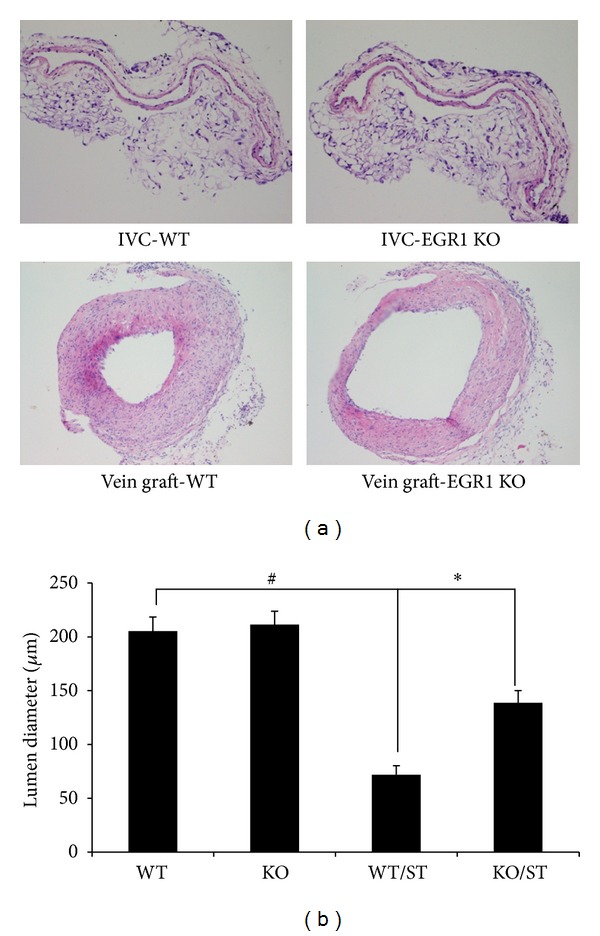
Egr-1 KO decreases lumen stenosis in vein grafts at 4 weeks after surgery. (a) Vein grafts from Egr-1 KO mice and WT mice were stained with hematoxylin and eosin (×100). (b) Quantitative analysis of lumen of vein grafts stenosis using NIS Elements BR software (NIKON, Japan). Data are expressed as mean ± SEM. *P < 0.05 versus WT/ST group; ^#^ *P* < 0.05 versus WT group. WT: inferior vena cava (IVC) in wild-type mice; WT/ST: vein graft from WT mice; Egr-1 KO: IVC in Egr-1 knockout mice; Egr-1 KO/ST: vein graft from Egr-1 knockout mice.

To demonstrate the pathophysiological importance of mechanical stretch-activated Egr-1, we construct mouse vein graft model in Egr-1 KO mice and WT mice. We harvested the vein grafts and analyzed them by HE staining at 4 weeks after surgery. As shown in Figure 1, the lumens of vein grafts in WT and Egr-1 KO mice were both narrower, compared with the lumen of inferior vena cava in WT mice (71.85 ± 8.36 *μ*m versus 205.25 ± 13.12 *μ*m, 138.73 ± 11.24 *μ*m versus 205.25 ± 13.12 *μ*m; *P* < 0.05). However, the lumen of vein grafts in Egr-1 KO mice was wider almost two fold, compared with that in WT mice (138.73 ± 11.24 *μ*m versus 71.85 ± 8.36 *μ*m; *P* < 0.05).

### 3.2. Egr-1 KO Inhibits ECs Proliferation Induced by Mechanical Stretch Stimulation

**Figure 2:**
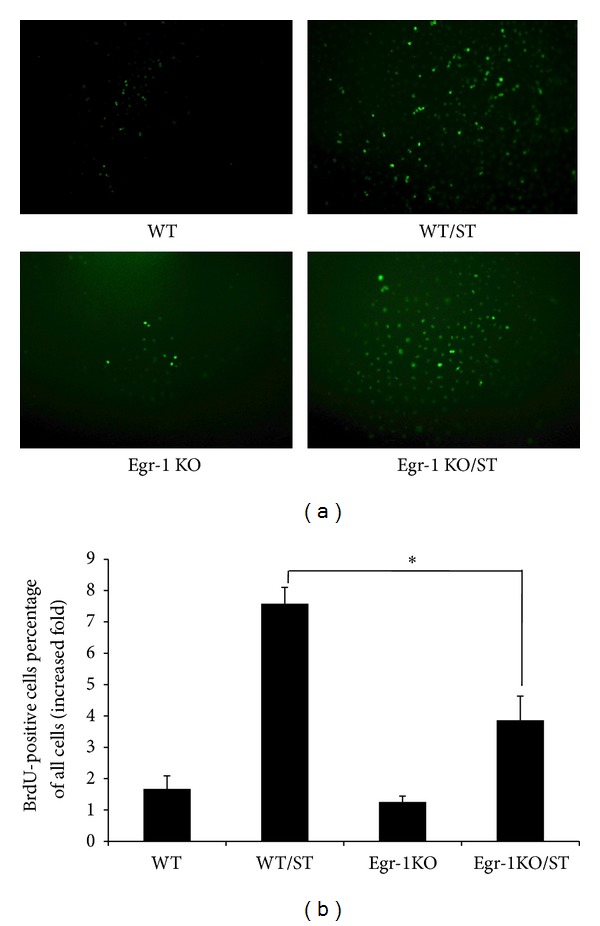
Egr-1 KO inhibits ECs proliferation induced by mechanical stretch stimulation. ECs were isolated from WT (above) and Egr-1 KO (below) mice. The ECs were submitted or not to mechanical stretch for 24 h, and BrdU staining was performed 24 h later. Cells were counted in 4 or 5 different views (×100) under fluorescence microscope and repeated 4 times. Data are expressed as mean ± SEM. *P < 0.05 versus WT/ST group. WT: wild-type mice; WT/ST: venous ECs from WT mice stimulated with mechanical stretch; Egr-1 KO: Egr-1 knockout mice; Egr-1 KO/ST: venous ECs from Egr-1 knockout mice stimulated with mechanical stretch.

To study the link between Egr-1 KO and mechanical stretch-induced ECs proliferation, we isolated ECs from veins of WT and Egr-1 KO mice. As shown in Figure 2, after mechanical stretch stimulation for 24 h, BrdU-positive cells in WT/ST ECs were increased by 7.6-fold compared with WT. However, the proliferation was suppressed in Egr-1 KO cells (50.9 ± 7.9% of WT/ST; *P* < 0.05).

### 3.3. Mechanical Stretch Increases Egr-1 Expression

**Figure 3:**
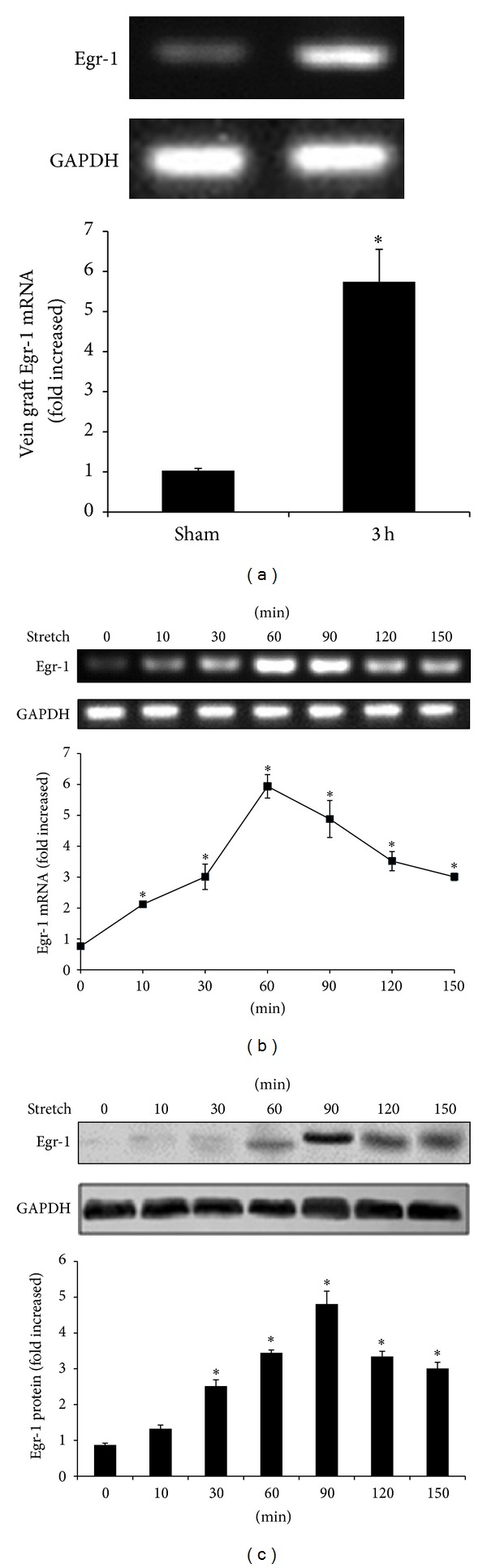
Mechanical stretch increased Egr-1 expression in wild-type (WT) mice. (a) Egr-1 mRNA levels in endothelial cells (ECs) increased 3 h after grafting the vein in WT mice (*n* = 5). *P < 0.05 versus sham group. Time course of Egr-1 mRNA (b) and protein (c) expression in ECs from WT mice after mechanical stretch (60 cycles/min and 15% elongation) for 0 to 150 min (*n* = 5). GAPDH was used as an internal control. Data are expressed as mean ± SEM. *P < 0.05 versus 0 min.

**Figure 4:**
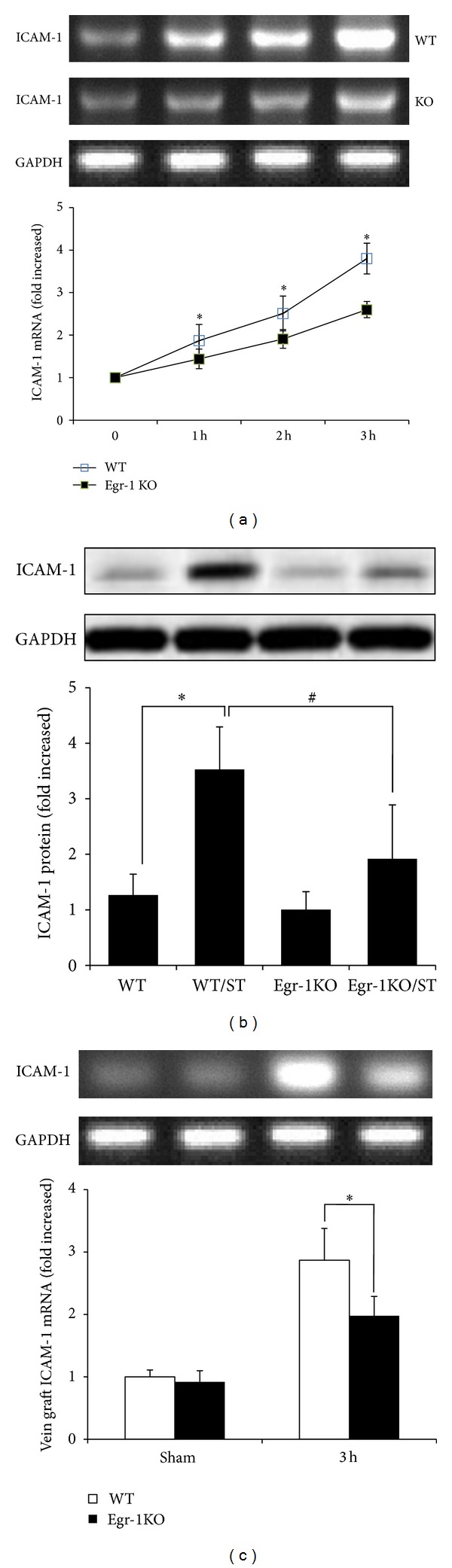
Egr-1 knockout (KO) decreased ICAM-1 expression. (a) Venous ECs from WT and Egr-1 KO mice were isolated and stimulated with mechanical stretch from 0 to 3 h (*n* = 5). ICAM-1 mRNA expression was determined by real-time RT-PCR. (b) Egr-1 KO decreased ICAM-1 protein levels after mechanical stretch stimulation for 24 h (*n* = 5). Data are expressed as mean ± SEM. *P < 0.05 versus WT group; ^#^ *P* < 0.05 versus WT/ST group. (c) Egr-1 KO decreased ICAM-1 mRNA expression in mouse vein graft model (*n* = 5). Data are expressed as mean ± SEM. *P < 0.05 versus WT group. WT: wild-type mice; WT/ST: venous ECs from WT mice stimulated with mechanical stretch; Egr-1 KO: Egr-1 knockout mice; Egr-1 KO/ST: venous ECs from Egr-1 knockout mice stimulated with mechanical stretch.

Vein grafts were harvested to measure Egr-1 mRNA levels. Three-h after being placed into carotid artery in WT mice, ECs isolated from veins showed significantly increased Egr-1 mRNA levels, compared with that in the sham surgery group (5.7 ± 1.6 fold; *P* < 0.05) (Figure 3(a)). The time course of Egr-1 mRNA expression in ECs from WT mice with mechanical stretch stimulation was assessed. As early as 10 min after being stimulated, Egr-1 mRNA levels increased and reached a peak at 60 min (5.9 ± 0.6 fold versus 0 min; *P* < 0.05). Egr-1 mRNA returned to baseline after 90 min (Figure 4(b)). Egr-1 protein reached a peak at 90 min (5.5 ± 0.5 fold versus 0 min; *P* < 0.05) (Figure 4(c)).

### 3.4. Egr-1 KO Suppressed ICAM-1 Expression

**Figure 5:**
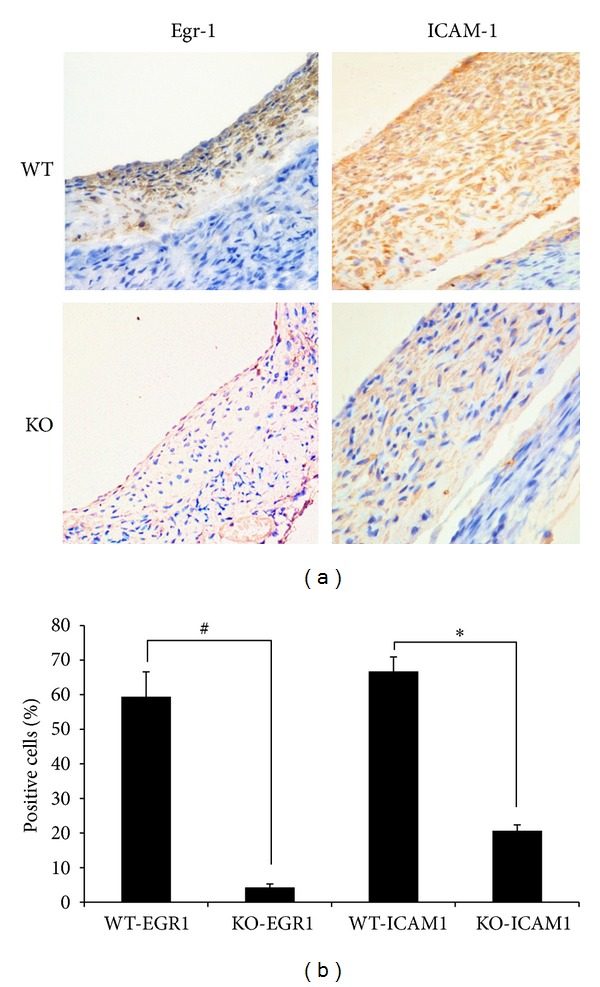
Immunohistochemistry of Egr-1 and ICAM-1 was performed in vein grafts from Egr-1 KO mice and WT mice at 4 weeks after surgery. (a) Vein grafts from Egr-1 KO mice and WT mice were stained with Egr-1 and ICAM-1 monoclonal antibody (×200). Brown colour indicates positive staining for all primary antibodies. (b) Quantitative analysis of the percentages of Egr-1 or ICAM-1 positive cells in vein grafts using NIS Elements BR software (NIKON, Japan). Data are expressed as mean ± SEM. ^#^ *P* < 0.05 WT-EGR1 versus KO-EGR1 group; *P < 0.05 WT-ICAM1 versus KO-ICAM1 group. WT-EGR1: vein grafts from WT mice were stained with Egr-1 monoclonal antibody; KO-EGR1: vein grafts from Egr-1 KO mice were stained with Egr-1 monoclonal antibody; WT-ICAM1: Vein grafts from WT mice were stained with ICAM-1 monoclonal antibody; KO-ICAM1: Vein grafts from Egr-1 KO mice were stained with ICAM-1 monoclonal antibody.

ICAM-1 plays an important role in inflammation after vascular injury [13]. We studied the role of Egr-1 in ICAM-1 expression. Venous ECs from WT and Egr-1 KO mice were isolated and stimulated with mechanical stretch from 0 to 3 h. After 3 h, mechanical stretch increased ICAM-1 mRNA expression in WT ECs and was significantly suppressed in ECs from Egr-1 KO mice (68.2 ± 8.2% of WT/ST; *P* < 0.05) (Figure 4(a)). ICAM-1 protein levels in ECs from Egr-1 KO mice were significantly reduced, compared with that in ECs from WT mice, after mechanical stretch stimulation for 24 h (54.3 ± 9.1% of WT/ST; *P* < 0.05) (Figure 4(b)). Subsequently, we explored whether Egr-1 regulate ICAM-1 in mouse vein graft model. We harvested vein grafts from WT and Egr-1 KO mice 3 h after surgery. In vein grafts from WT mice, ICAM-1 mRNA was significantly increased, compared to WT mice in the sham surgery group (2.9 ± 0.9 fold; *P* < 0.05). Egr-1 KO significantly decreased ICAM-1 expression (61.0 ± 10.3% of vein grafts from WT mice 3 h after surgery; *P* < 0.05) (Figure 4(c)). Immunohistochemistry showed that the percentages of Egr-1- and ICAM-1- positive cells both reduced in vein grafts from Egr-1 KO mice at 4 weeks after surgery, compared to that from WT mice (4.3 ± 1.0% versus 59.4 ± 7.2%, 20.7 ± 1.7% versus 66.7 ± 4.2%; *P* < 0.05) (Figure 5).

## 4. Discussion

In this study, we found that the expression of Egr-1 in endothelial cells stimulated by mechanical stretch was significantly increased and the ECs proliferation activity was significantly improved. We also found that Egr-1 mRNA expression changed over time; it increased after mechanical stretch stimulation for ten minutes, reached a peak after stimulation for 60 min, and then gradually declined. In the early stage after CABG surgery, the vein graft was under high arterial blood pressure; then the sudden hemodynamic change and mechanical stretch stimulation could damage endothelial cells. The increase of Egr-1 mRNA expression after stimulation for ten minutes indicated that Egr-1 was expressed in endothelial cells immediately after the cells were damaged, which subsequently induced a series of pathophysiological changes. Egr-1 belongs to the immediate early gene rather than the long-term gene. Apparently, its expression was not sustained as opposed to long-term gene expression. Hence, Egr-1 mRNA expression declined after reaching a peak. It was reported that the duration of Egr-1 expression varied with different stimulations [34–36]. When Egr-1 expression was upregulated, it could be regulated by negative feedback regulators such as c-fos [37], phosphatase inhibitors [38], and free radical scavengers [39].

After mechanical stretch stimulation, the expression of Egr-1 and ICAM-1 in ECs cells of WT mice was significantly increased, whereas expression of ICAM-1 in ECs cells or vein graft of Egr-1 KO mice was significantly inhibited in this study. Meanwhile, the intimal hyperplasia was greatly reduced in the vein graft of Egr-1 KO mice. It suggested that Egr-1 expression could upregulate ICAM-1 expression, increase inflammatory cell adhesion, aggravate vascular inflammation, and result in vein graft restenosis. However, Egr-1 knockout did not completely inhibit inner cortex thickening and intimal hyperplasia indicating that other factors may be involved in the process of intimal hyperplasia. It was reported that mechanical strain can enhance NF-*κ*B activity and NF-*κ*B can increase intimal hyperplasia and result in restenosis by regulating IGF-1R transcription [29].

In addition to mechanical strain stimulation, vein graft was also subjected to oxidative stress injury. Oxidative stress-induced apoptosis was a key to a variety of cardiovascular diseases and it was found throughout intimal hyperplasia after vascular injury was induced by a variety of damaging factors [40]. After CABG surgery, the vein graft, especially the anastomosis, was infiltrated by macrophages and covered with lipid deposition [41]. The macrophages activated by inflammation could produce excessive reactive oxygen species (ROS), and increase interleukin-8 (IL-8) and monocyte chemotactic protein-1 (MCP-1), which aggravated inflammation again [42]. The ischemia-reperfusion reaction during surgery not only produced excessive ROS, resulting in oxidative damage for the vascular wall cells, but also upregulated Egr-1 expression directly [43]. ROS could upregulate Egr-1 expression by activating families of protein kinase K isoenzymes and the mitogen-activated protein kinases/extracellular signal-regulated kinases (MEK/ERK) pathway [44], and increase lipid deposition in the blood vessel wall. The deposited low-density lipoprotein (LDL) in the ROS could form oxidized LDL (ox-LDL) protein, stimulate VSMC proliferation by activating caspase family proteins and mediating the NF-*κ*B signaling pathway, and eventually lead to intimal hyperplasia and stenosis [45].

In summary, Egr-1 played an important role in vein graft restenosis. It could lead to vein graft restenosis probably by upregulating ICAM-1, aggravating vascular inflammation, and promoting endothelial cell proliferation. As a key factor of vein graft restenosis, Egr-1 may be used for an effective target for gene prevention and treatment.

## 5. Conclusion

Our results suggest that Egr-1 plays a role in restenosis by upregulating ICAM-1 in ECs in vein graft, which may provide new clues for the prevention of vein grafts restenosis in CABG, leading to new strategies to improve patients' prognosis after CABG. However, the specific mechanisms still need to be elucidated, as well as the exact relations between ECs and SMCs during restenosis.
