# Corrigendum to “Early Growth Response Protein 1 Promotes Restenosis by Upregulating Intercellular Adhesion Molecule-1 in Vein Graft”

**DOI:** 10.1155/2021/1297378

**Published:** 2021-01-27

**Authors:** Kui Zhang, Jian Cao, Ran Dong, Jie Du

**Affiliations:** ^1^Cardiac Surgery, Beijing Institute of Heart, Lung and Blood Vessel Diseases, Beijing Anzhen Hospital Affiliated with Capital Medical University, Beijing 100029, China; ^2^Vessel Biology, Beijing Institute of Heart, Lung and Blood Vessel Diseases, Beijing Anzhen Hospital Affiliated with Capital Medical University, Beijing 100029, China

In the article titled “Early Growth Response Protein 1 Promotes Restenosis by Upregulating Intercellular Adhesion Molecule-1 in Vein Graft” [1], there was an error in Figure 4(c). The figure should show the internal control “GAPDH” blot. The corrected figure is shown below and is listed as Figure 4.

## Figures and Tables

**Figure 1:**
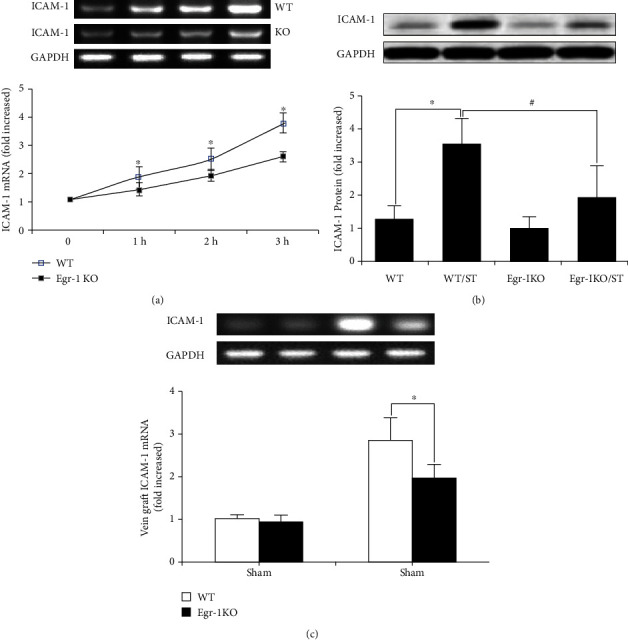
Egr-1 knockout (KO) decreased ICAM-1 expression. (a) Venous ECs from WT and Egr-1 KO mice were isolated and stimulated with mechanical stretch from 0 to 3 h (*n* = 5). ICAM-1 mRNA expression was determined by real-time RT-PCR. (b) Egr-1 KO decreased ICAM-1 protein levels after mechanical stretch stimulation for 24 h (*n* = 5). Data are expressed as mean ± SEM. ∗*P* < 0.05 versus the WT group; ^#^*P* < 0.05 versus the WT/ST group. (c) Egr-1 KO decreased ICAM-1 mRNA expression in the mouse vein graft model (*n* = 5). Data are expressed as mean ± SEM. ∗*P* < 0.05 versus the WT group. WT: wild-type mice; WT/ST: venous ECs from WT mice stimulated with mechanical stretch; Egr-1 KO: Egr-1 knockout mice; Egr-1 KO/ST: venous ECs from Egr-1 knockout mice stimulated with mechanical stretch.
